# Immunoglobulin-mediated immune dysregulation in recurrent pregnancy loss: mechanisms, biomarkers, and therapeutic perspectives

**DOI:** 10.1007/s12026-026-09820-z

**Published:** 2026-08-17

**Authors:** Kamala C, Pallavi Shekar, Umme Kulsum, Ananya Hegde, Lakshitha V Jayakrishna, Deepa Bhat, Meghana Hangala Shivakumaraswamy, Akila Prashant, Praveen Kumar K S

**Affiliations:** 1 Department of Medical Genetics, JSS Medical College and Hospital, JSS Academy of Higher Education & Research, Mysuru, 570015 Karnataka India; 2 Department of Biochemistry, JSS Medical College and Hospital, JSS Academy of Higher Education & Research, Mysuru, 570015 Karnataka India; 3 Department of Anatomy, JSS Medical College and Hospital, JSS Academy of Higher Education & Research, Mysuru, 570015 Karnataka India; 4 Department of Obstetrics and Gynaecology, JSS Medical College and Hospital, JSS Academy of Higher Education & Research, Mysuru, 570015 Karnataka India; 5 Special Interest Group-Translational Research in Regenerative Genetics (SIG-TRRG), JSS Medical College and Hospital, JSS Academy of Higher Education & Research, Mysuru, 570015 Karnataka India

**Keywords:** Recurrent pregnancy loss, Immunoglobulins, Autoantibodies, Maternal–fetal tolerance, Placental dysfunction, Intravenous immunoglobulin (IVIG)

## Abstract

Recurrent pregnancy loss (RPL) is a multifactorial reproductive disease that is typified by repeated pregnancy loss with immune dysregulation emerging as a critical contributing factor. Immunoglobulins are among the immune components that are instrumental in preserving tolerance between the mother and the fetus, regulating inflammatory responses, and supporting placental development. This review provides a comprehensive overview of the role of immunoglobulins in recurrent pregnancy loss, with particular emphasis on immune dysregulation, emerging biomarkers, and therapeutic implications. The immunoglobulin profiles, such as the changes in IgG subclasses and the presence of pathogenic autoantibodies, e.g. antiphospholipid and antinuclear antibodies, are highly correlated with unfavourable pregnancy outcomes. These changes, in a mechanistic manner, lead to poor trophoblast invasion, endothelial dysfunction, thrombosis and cytokine imbalance. Emerging evidence also highlights the contribution of regulatory B cells and the antibody-mediated immune network in maintaining pregnancy homeostasis. Immunoglobulin profiling has clinical potential as a diagnostic and prognostic methodology that can be used to stratify patients and provide customised therapy. Immunomodulatory therapies, especially intravenous immunoglobulin (IVIG), are potentially effective in a limited range of patient populations, but clinical response is variable. On the whole, this review demonstrates the role of immunoglobulin-mediated mechanisms in RPL and their future potential in enhancing precision medicine approaches to better diagnosis and management.

## Introduction

The primary characteristic of recurrent pregnancy loss (RPL) is repeated pregnancy failures, which is associated with significant emotional, psychological, and clinical burden among affected couples [1, 2]. Recurrent pregnancy loss (RPL) is defined differently by major international professional societies [3]. The American Society for Reproductive Medicine (ASRM) defines RPL as two or more failed clinical pregnancies and recommends evaluation after two losses [4]. Similarly, the European Society of Human Reproduction and Embryology (ESHRE) defines recurrent pregnancy loss as two or more pregnancy losses and recommends clinical evaluation after two losses, irrespective of whether they are consecutive [5]. In contrast, the Royal College of Obstetricians and Gynaecologists (RCOG) traditionally defines recurrent pregnancy loss as three or more consecutive pregnancy losses [6]. These differences contribute to variations in the reported prevalence of RPL and the timing of clinical investigation [7]. Early evaluation after two pregnancy losses may facilitate timely identification of underlying etiologies and improve reproductive management [8]. While the actual rate of occurrence is influenced by differing definitions of RPL, as well as by various population characteristics, studies have estimated that the current prevalence of RPL globally is 1–2% [9, 10]. Although RPL is considered a unique clinical condition with potentially multiple etiologies, women are more likely to experience an isolated miscarriage, as these occur in approximately 15–20% of all clinically confirmed pregnancies [11, 12]. Maternal age and history of previous miscarriages, as well as underlying health and genetic conditions, are all factors related to an increased likelihood of pregnancy loss [10, 13], which also highlights the complex relationship between maternal, fetal and placental factors necessary to support a successful pregnancy [14]. Despite significant advances in reproductive medicine and diagnostic technologies, approximately 40–50% of recurrent pregnancy loss (RPL) cases remain unexplained even after comprehensive clinical evaluation [9, 15]. The known etiologies of RPL include chromosomal abnormalities, congenital or acquired uterine anomalies, endocrine disorders, inherited or acquired thrombophilia, and immune-mediated dysfunction [9, 16]. Among these etiologies, immune dysregulation has attracted increasing attention because successful pregnancy depends on the establishment and maintenance of maternal–fetal immune tolerance [17, 18]. Failure to establish adequate immune tolerance toward the semi-allogeneic fetus may result in impaired implantation, abnormal placentation, and recurrent pregnancy loss [19, 20]. Therefore, a better understanding of the immunological mechanisms underlying RPL may improve disease characterization, facilitate biomarker discovery, and support the development of targeted therapeutic strategies [21, 22]. Among the various immune components implicated in RPL, immunoglobulins have emerged as important mediators because of their roles in autoantibody production, complement activation, Fc receptor signalling, and regulation of maternal–fetal immune tolerance [23]. The definition of recurrent pregnancy loss varies among professional organizations [3]. The American Society for Reproductive Medicine (ASRM) defines RPL as two or more failed clinical pregnancies, whereas the European Society of Human Reproduction and Embryology (ESHRE) also supports evaluation after two pregnancy losses [24]. In contrast, the Royal College of Obstetricians and Gynaecologists (RCOG) traditionally defines recurrent pregnancy loss as three or more consecutive pregnancy losses [25]. Throughout this review, the term recurrent pregnancy loss (RPL) is used consistently while acknowledging these differences in international definitions [3].

### Challenges in the current classification of RPL

 The existing methodology for classifying RPL is primarily founded upon such identifiable factors as anatomical abnormality, genetic predisposition, an imbalance of hormones, the presence of infectious organisms, the ability to form an abnormal blood clot, or the presence of autoantibodies [9, 26]. Although this approach has provided structure for diagnosing and managing recurrent pregnancy loss, it has many drawbacks that inhibit a complete picture of the causes of recurrent pregnancy loss [15, 27]. Despite undergoing extensive diagnostic evaluation, a significant percentage of cases continue to be undiagnosed, which identifies the limitations of traditional clinical methodology [9, 28]. This implies that currently used classification systems do not adequately consider the subtle underlying molecular, immunological, and pathophysiological mechanisms required for continued support of a viable pregnancy [21, 29]. Also, traditional classifications frequently highlight structural or laboratory-identifiable abnormalities and do not pay much attention to dynamic immune system interactions between mother and fetus [20, 22]. For the pregnancy to progress normally, there must be a tightly controlled coordination of innate with adaptive immune responses, and traditional evaluations of clinical immunology commonly rely on very few immune system markers, which limit our knowledge about the nature of the immune dysfunction associated with recurrent pregnancy loss [17, 18]. For example, abnormal immunoglobulin profiles, altered Fc receptor signaling, or malfunctioning antibody-mediated immunological tolerance mechanisms may not be detected by standard testing [30, 31]. Furthermore, etiological (causal) categories have a high degree of overlap. Areas such as endocrine (hormonal) disorders can affect how well the immune system works, or genetic factors can alter the way the immune system regulates itself [32, 33]. The interrelationships between these different etiological categories are not well integrated into the current classification systems, creating more fragmentation than integration in the management process [27, 34]. Furthermore, the majority of classifications lack molecular insights that could direct targeted medicines because they are based on observational data [29, 35].

### Immunoglobulins as key mediators in RPL pathogenesis

Immunoglobulins have a pivotal function in promoting humoral immunity, maternal and fetal tolerance through their usage during frequent miscarriages [36, 37]. The combination of the semi-allogenic characteristics of the fetal tissue and the need to protect a mother from disease creates an environment that an immunologically competent immune system must recognize and adapt to achieve a successful pregnancy [19, 32]. Immunoglobulins are an important part of the immune system and can be used for different tasks such as regulating the immune response, recognizing antigens, and controlling inflammation [38]. Recent studies have provided evidence that atypical immunoglobulin levels and/or abnormal specificities of antibodies, as well as dysregulated Fc-mediated signaling, could be related to poor outcomes of pregnancy [30, 31]. The best characterized immunological basis of RPL is antiphospholipid antibodies; however, recent findings indicate that there are also non-classical autoantibodies (particularly anticardiolipin, anti-beta-2-glycoprotein-I, and others) which affect reproductive success in the way mentioned above [39, 40]. These additional types of autoantibodies can interfere with trophoblast function and/or promote the activation of complement pathways and/or disturb normal placentation development [38, 41]. Immunoglobulins also modulate cytokine networks, affect the interactions of the immune system cells with each other, and help maintain the proper function of the placenta. IVIG therapy has gained interest because of its immunomodulating activity, including the ability to suppress inflammatory cytokines, neutralize pathogenic antibodies, and modulate Fc receptor signaling [41, 42]. However, the effectiveness of IVIG therapy in the clinical setting has been variable, highlighting the need for improved patient selection and understanding of the mechanisms responsible for clinical success or failure [43, 44]. A deeper understanding of immunoglobulin-mediated mechanisms will enhance diagnostic accuracy and enable the development of targeted immunotherapies, ultimately improving personalized treatment strategies for RPL [29, 30]. Besides enhancing diagnostic clues, knowledge on how immunoglobulins regulate immunological tolerance, placental activity and inflammatory homeostasis can enable specific immunotherapies to be achieved [45, 46]. The findings will be critical in enhancing individualized medicine approaches to treat RPL [26].

### Scope and novelty of the review

Despite many advances in reproductive immunology, the current review articles are mainly dedicated to the study of some single aspect of recurrent pregnancy loss (RPL), such as APS, maternal-fetal immune tolerance, particular autoantibodies, or the use of intravenous immunoglobulin (IVIG) therapy [47]. While all these reviews provided us with much useful information about immune-mediated pregnancy loss, they usually consider immunoglobulin biology only as one of the parts of a more complicated immunological system [48]. The synthesis of the available data that would be devoted exclusively to immunoglobulin-mediated immune dysregulation and its role in the pathology, diagnostics and treatment of RPL is missing [49].

This review aims to fill the above-mentioned knowledge gap and provides an evidence-based summary of different functions of immunoglobulins in recurrent pregnancy loss. Besides the description of already known mechanisms, which involve antiphospholipid antibodies and obstetric APS, this review critically analyzes recent findings about IgG subclasses, Fc glycosylation, Breg malfunction, complement activation, and non-criteria autoantibodies [50]. Moreover, the discussion of different methods of therapy based on the antibody-mediated immune system is included here, taking into account available clinical evidence [48].

Significantly, in this review, the distinction is made between the established mechanism and biomarker on the one hand and promising diagnostic biomarker and therapy on the other; hence, the balance in evaluating the existing body of evidence is achieved and key gaps in our knowledge and directions for future research are identified [51]. The combination of molecular immunology with translational and clinical aspects of the issue at hand allows achieving an all-encompassing approach in investigating the immunoglobulin-mediated immune dysregulation [52].

### Literature search strategy

This review was conducted as a narrative review to comprehensively summarize the current evidence on immunoglobulin-mediated immune dysregulation in recurrent pregnancy loss (RPL) [53]. A systematic literature search was performed using PubMed/MEDLINE, Scopus, Web of Science, Embase, and Google Scholar to identify relevant studies published between January 2005 and May 2026. The search strategy included combinations of the following keywords: “recurrent pregnancy loss,” “recurrent miscarriage,” “immunoglobulins,” “IgG subclasses,” “autoantibodies,” “antiphospholipid syndrome,” “complement activation,” “Fc glycosylation,” “regulatory B cells,” “intravenous immunoglobulin,” “immune tolerance,” and “reproductive immunology [47].”

Priority was given to systematic reviews, meta-analyses, randomized controlled trials, international clinical practice guidelines (ASRM, ESHRE, and RCOG), and high-quality observational studies [49]. Additional relevant publications were identified through manual screening of reference lists from selected articles. Studies were included based on their relevance to the immunological mechanisms, biomarkers, and therapeutic implications of immunoglobulin-mediated immune dysregulation in RPL [48]. The evidence was critically synthesized to distinguish established clinical findings from emerging biomarkers and experimental therapeutic approaches [47].

## Immunological adaptations in normal pregnancy

### Mechanisms of maternal–fetal immune tolerance

The immune system of the mother must create ideal conditions to allow the growing fetus (which is half genetically different from its mother) to grow continuously, yet still have the capacity to protect itself from infection [32]. Immune tolerance (to the fetus) is not an immune system that is shut down [54]. Rather, an immune system tolerant of a fetus is very regulated and appropriately functioning. Immune cell(s), trophoblasts, and soluble mediators are all integral components of this process and must work in conjunction with one another [55]. At the maternal–fetal interface, humoral immunity is a key player, and this type of immunity depends on immunoglobulins [37]. Immunoglobulins are important for regulating the immune system as well as providing protection from infections and helping with tissue remodeling [56]. IgG antibodies are especially important at the maternal–fetal interface because they can bind to Fc receptors that are found on the surface of trophoblasts, decidual macrophages, and uNK cells [57]. The binding of IgG to these cells can assist in modulating the immune response at the maternal–fetal interface and transferring of maternal antibodies across the placenta. These interactions influence the cellular immunological responses, complement activation, and cytokine profiles, all of which play a vital role in placental growth and implantation [36].

### Humoral immune regulation at the maternal–fetal interface

During pregnancy, humoral immunity has a protective function, neutralizing pathogens as well as preventing an overzealous inflammatory response that would be detrimental to a developing fetus [58]. The role of immunoglobulin is to assist in establishing an immune milieu that supports fetal development [36]. An alteration in the production/function of antibodies can create an imbalance, leading to an overactive immune response [31]. This alteration of antibody levels can manifest as the presence of autoantibodies, improper distribution of IgG subclasses (abnormal levels), and/or excessive complement activity within tissue that is trophoblast trophic, at the maternal-fetal interface, which can impair trophoblast invasion and/or disrupt placental vascular development and/or create inflammation at the maternal-fetal interface site. Such immunological disturbances have been increasingly associated with recurrent pregnancy loss, particularly in cases where other causes are not identified [59].

### Protective versus pathogenic immune responses in pregnancy

The success of maintaining a pregnancy relies heavily on the interactions of both protective immune response mechanisms and those that may lead to negative outcomes [32]. The role of immunoglobulins in these processes has both a positive and a negative impact [56]. They will aid in developing an immune tolerance while also offering protection against infection [36]. Conversely, when there is a lack of regulation with immunoglobulins, they may play a part in developing an immune-mediated complication during pregnancy [31]. The effectiveness of immunoglobulin function is influenced by factors like their specificity, concentration, subclass distribution, and the manner in which they interact with immune cells [38]. Disruption in these parameters could result in inflammatory changes, impaired development of the placenta, and miscarriage [60]. Understanding the dual role of immunoglobulins is an important step in finding therapeutic targets to restore immune balance in women experiencing recurrent pregnancy loss [30].

## Immunoglobulins: Structure, classes, and biological functions

Immunoglobulins are important parts of the humoral immune system and are said to contribute significantly to the maintenance of immunological balance during pregnancy [36]. Immunoglobulins can be categorized into four different types based on their different structures, distribution, and biological functions: IgG, IgM, IgA, and IgE [61]. IgG accounts for the greatest percentage of circulating immunoglobulins and has been shown to function largely as an immunoregulatory agent and as the primary agent of opsonization and long-term immunity [62]. The initial immune response leads to the production of IgM as the primary antibody and is indicative of acute or recent antigen exposure [63]. The structure of IgM as a pentamer prevents it from crossing the placenta [37]. IgA is primarily located in mucosal secretions and contributes to immunity at epithelial surfaces, such as those lining the reproductive tract [64]. IgE, despite its low concentration, participates in allergic and inflammatory reactions by activating mast cells and basophils [65]. IgD, although one of the five major immunoglobulin isotypes, is primarily expressed as a membrane-bound receptor on naïve B lymphocytes and contributes to B-cell activation and maturation [66]. However, current evidence supporting a direct role for IgD in the immunopathogenesis of recurrent pregnancy loss is limited; therefore, this review focuses primarily on immunoglobulin classes for which stronger experimental and clinical evidence is available [47]. An adequate balance of production and functionality of all immunoglobulin main classes is required for maintaining maternal immunological tolerance and protecting against infection during pregnancy [54]. Dysregulation of immunoglobulin levels or activities has been linked with negative consequences for pregnancy, including recurrent pregnancy loss [40].

### Placental transfer and functional diversity of IgG subclasses

IgG can be classified into four different subclasses (IgG1, IgG2, IgG3 and IgG4) with varying properties for activation of the immune response [62]. IgG1 and IgG3 are very effective at activating complement and binding to Fc receptors, resulting in the ability to activate different immune effector functions [38]. Conversely, IgG4 is considered to have anti-inflammatory effects based on its reduced capability to activate complement [67]. The maternal IgG is transferred to the fetus via the neonatal Fc receptor (FcRn) found on syncytiotrophoblast cells [58]. This transfer process during pregnancy is selective; transfer increases to a greater extent in T2 and T3 [36]. Antenatal IgG transfer provides passive immunity; however, it could also facilitate the transmission of pathogenic antibodies, possibly resulting in pregnancy complications [68].

### Biological functions of immunoglobulins in pregnancy

Altered distribution of IgG subclass or autoantibodies might contribute to miscarriage, trophoblast dysfunction, placental dysfunction, and loss of maternal-fetal immunologic tolerance. Abnormal IgG subclass pattern and reduced efficiency of placental transfer pathways have been established as contributory factors to the immunological aspects of RPL in women who have experienced multiple losses [57]. The major classes of immunoglobulins, their biosynthetic roles during pregnancy, and their clinical relevance in RPL are described in Table 1.

**Table 1:** Overview of immunoglobulin classes and their functional roles in pregnancy

Immunoglobulin	Structural Features	Key Immunological Function	Role in Normal Pregnancy	Dysregulation in RPL	Clinical Utility / Biomarker Potential
IgG	Monomer; subclasses differ in Fc function and complement activation	Opsonisation, complement activation, Fc receptor signalling, and immune regulation	Active placental transfer via FcRn promotes fetal immunity and immune tolerance	Altered subclass distribution (↓IgG4, ↑IgG1/IgG3), pathogenic autoantibodies, abnormal Fc glycosylation	Key biomarker in immune RPL; target for IVIG therapy; subclass profiling improves risk stratification [62]
IgM	Pentamer; high avidity; large size prevents placental transfer	Primary immune response, complement activation	Indicates acute immune activation; does not cross the placenta	Elevated levels may reflect infection or immune activation contributing to miscarriage	Marker of recent infection; supportive diagnostic indicator [63]
IgA	Monomer/dimer; predominant in mucosal secretions	Mucosal immunity, immune exclusion, and anti-inflammatory effects	Maintains reproductive tract immune homeostasis	Reduced mucosal immunity may predispose to inflammatory responses	Potential marker for mucosal immune dysfunction (emerging evidence) [64]
IgE	Monomer; binds mast cells and basophils	Allergy, inflammatory mediator release	Limited role in normal pregnancy	Elevated levels may contribute to an inflammatory milieu and an immune imbalance.	Limited clinical utility; exploratory role in immune dysregulation exploratory role in immune dysregulation. [65]

**Fig. 1:**
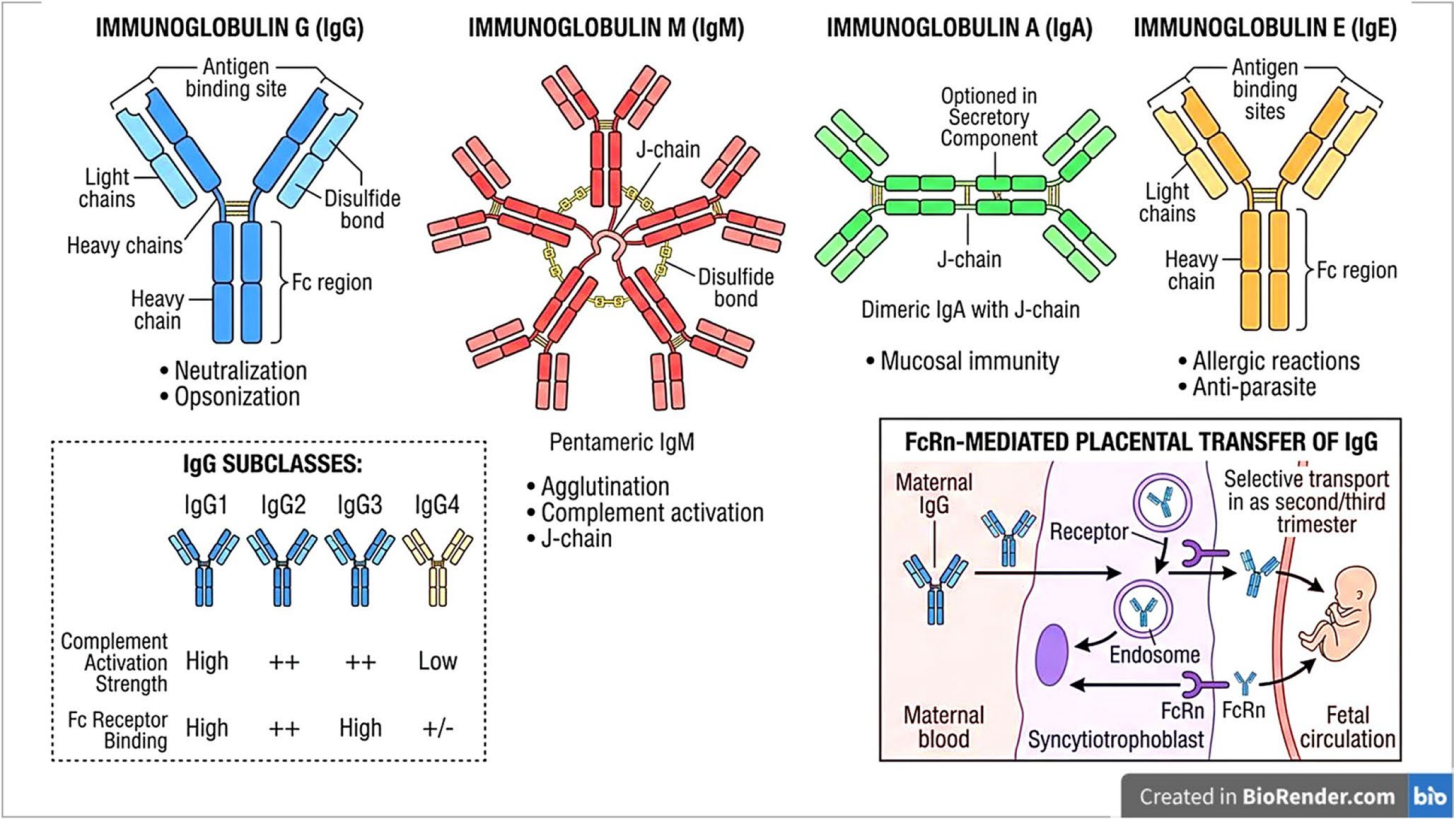
Overview of immunoglobulin classes and FcRn-mediated placental IgG transfer

Immunoglobulins play a complicated role in ensuring immunological balance during pregnancy by regulating both innate and adaptive immune responses [56]. The immunoglobulins influence the invasion of the trophoblast, placental growth, and immune cell activity locally at the mother-fetal junction [69]. Protective immunoglobulins regulate the natural killer cell cytotoxicity, macrophage polarization, and cytokine secretion, and this can result in immunological tolerance and establishment of a conducive environment that encourages pregnancy [56]. Conversely, pathogenic autoantibodies or excessive activation of complement via antibody can trigger inflammation, endothelial damage, and placental insufficiency [70]. An overview of the major immunoglobulin classes, their structural and functional characteristics, and the FcRn-mediated placental transfer of maternal IgG antibodies is presented in Fig. 1.

Overview of the major immunoglobulin classes (IgG, IgM, IgA, and IgE), highlighting their structural characteristics and principal immunological functions. The figure also illustrates the functional diversity of IgG subclasses (IgG1–IgG4) and the FcRn-mediated selective placental transfer of maternal IgG antibodies, an essential mechanism for passive fetal immunity and neonatal immune protection during pregnancy.

## Immunoglobulin-associated immune dysregulation in RPL

### Autoimmune-mediated pathways in RPL

Autoimmune processes have been identified as one of the leading causes of RPL [9]. Autoimmune dysregulation contributes to placental dysfunction and impaired fetal viability in RPL [69]. Antiphospholipid antibodies (aPL), which include lupus anticoagulant, anticardiolipin and anti-beta2-glycoprotein I, represent the largest body of evidence for risk due to autoimmunity [39]. These antibodies are linked with abnormal trophoblast invasion, inadequate remodeling of spiral arteries, thrombosis of the placenta and inflammatory changes [71]. In addition to classical antiphospholipid syndrome, research suggests that other types of autoantibodies, such as antinuclear, anti-thyroid, and anti-trophoblast, also play a role in RPL [72]. These autoantibodies may impair placental function and alter immune regulation at the maternal–fetal interface [69].

### Altered immunoglobulin production and B-Cell dysfunction

B-cells are important for normal pregnancy as their activity is regulated by cytokines and they produce protective immunoglobulins, particularly asymmetric IgG [73]. Studies have demonstrated that women with RPL have abnormalities in their B2 lymphocyte subpopulations, including fewer regulatory B (Breg) lymphocytes and more autoreactive B lymphocytes [74]. As a result, their immunoglobulin profile is altered, with increased autoantibody production and an imbalance of IgG subclasses [40]. Furthermore, reduced anti-inflammatory IgG4 levels and altered Fc glycosylation patterns may impair immunosuppressive signals at the maternal–fetal interface [75]. Additionally, abnormal signaling of B lymphocytes can amplify inflammatory responses, extending the risk of failure of fetal tolerance [76].

### Immunoglobulin-mediated inflammation and complement activation

Immune-driven pregnancy loss is largely due to complement activation and inflammation from antibodies [77]. Pathogenic immunoglobulins that bind with placental or endothelial antigens activate the classical complement pathway, resulting in injury and inflammation to the tissue [78]. The complement system produces C3a and C5a as inflammatory substances, activating immune responses by attracting immune cells and facilitating the loss of endothelial function in placental blood vessels [79]. Such mediators recruit immune cells and are stimulated to induce endothelial dysfunction in the placental vasculature. There is also a correlation between an over-activation of the complement system and a reduction in angiogenesis, placental insufficiency, and fetal growth restriction during recurrent pregnancy loss [46]. In addition, inflammation caused by immunoglobulins disturbs the balance between pro-inflammatory and anti-inflammatory signals necessary for normal implantation and maintenance of early pregnancy [69]. The major autoantibodies implicated in recurrent pregnancy loss, their immunological targets, and associated clinical effects are summarised in Table 2.

**Table 2:** Autoantibodies associated with recurrent pregnancy loss

Auto Antibody	Molecular Target	Pathogenic Mechanism	Impact on pregnancy	Clinical significance
Antiphospholipid antibodies (aPL)	Phospholipids, β2-glycoprotein I	Complement activation, endothelial dysfunction, thrombosis, and impaired trophoblast invasion	Placental thrombosis, defective implantation, fetal loss	Major immune-mediated cause of RPL [39].
Antinuclear antibodies (ANA)	Nuclear antigens	Immune complex formation, inflammation, and NK cell activation	Impaired implantation, placental inflammation	Associated with Unexplained RPL[80].
Anti-thyroid antibodies (TPO, Tg)	Thyroid proteins	Systemic autoimmune activation, immune imbalance	Increased miscarriage risk even in euthyroid women	Marker of generalized autoimmunity [72].
Anti-prothrombin / anti-protein S	Coagulation proteins	Prothrombotic state, impaired anticoagulation pathways	Microvascular thrombosis in the placenta	Non-criteria APS-related RPL [81].
Anti-annexin V / anti-HLA	Placental and immune regulatory proteins	Disruption of the anticoagulant shield, immune activation	Placental insufficiency, implantation failure	Emerging contributors to unexplained RPL [71].

## Pathogenic mechanisms of immunoglobulins in RPL

### Impaired trophoblast invasion and placentation

Immune imbalance at the maternal–fetal interface may impair trophoblast invasion and placental development, including invasion into the uterine lining and development of the placenta, all of which are crucial for creating a viable pregnancy [32]. Immunoglobulins can alter the ability of trophoblast to invade, migrate or discriminate, in particular autoantibodies, including antiphospholipid antibodies [69]. In trophoblastic cells, antibodies that are abnormally bound can disrupt the adhesion, the growth factor signal transduction pathway, as well as the interaction with the extracellular matrix [80]. These alterations contribute to defective spiral artery remodeling and placental insufficiency, leading to placental insufficiency and subsequently repeated pregnancy loss; this is defined as an immune-mediated placentation impairment [81].

### Endothelial dysfunction and thrombotic pathways

In order to have an appropriate amount of blood available to the developing fetus, it is important to keep the endothelium intact and maintain the normal process of coagulation within the body [82]. In addition, RPL is associated with endothelial cell activation and inflammation, in addition to a pro-thrombotic condition created by pathogenic immunoglobulins (i.e., antibodies), most notably antiphospholipid antibodies about recurrent pregnancy loss, both antibodies lead to platelet aggregation as well as the expression of tissue factors, thereby leading to the activation of the complement pathway and consequently form micro-thrombi in the placental vessels [71]. The resultant occlusion of the vascular supply to the placenta leads to ischemia, thereby threatening the delivery of oxygen and nutrients to the fetus, making the thrombo-immunologic pathways activated by the immunoglobulin critically important contributors to pregnancy loss [78]. The ability of immunoglobulins to play an important role in promoting placental growth and vascular remodeling is underscored by the critical importance of uterine natural killer (uNK) cells at the earliest stages of gestation [83]. These women may exhibit increased susceptibility to hyperimmune responses, impaired maternal–fetal tolerance, and disrupted implantation [59].

### Treg–Th17 imbalance and cytokine dysregulation

**Fig. 2:**
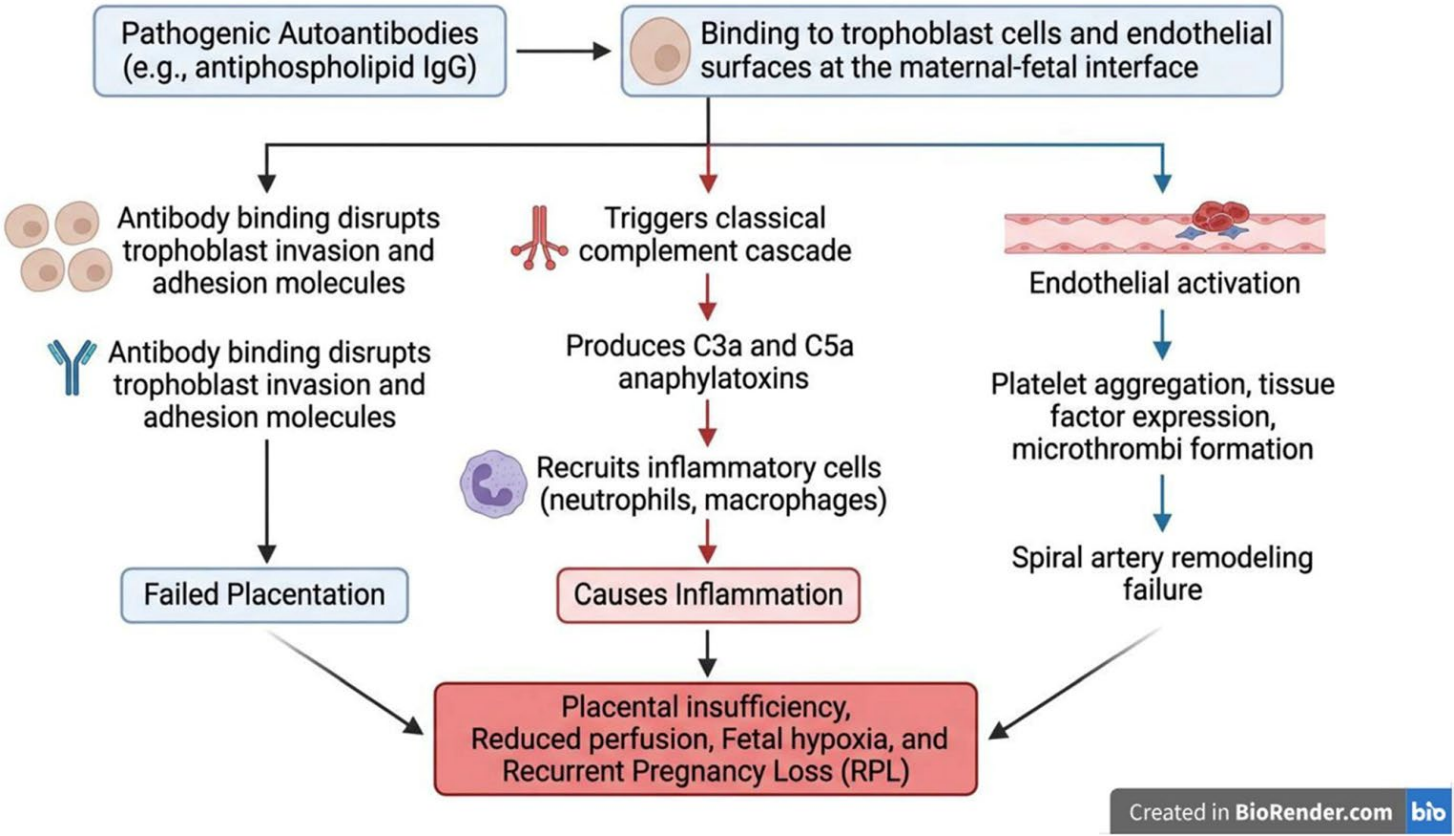
Immunoglobulin-mediated pathogenic mechanisms in recurrent pregnancy loss

To sustain maternal immune tolerance in pregnancy, there is a correct balance between regulatory T-cells and T-helper [17] cells [54]. The balance between these two is altered by the action of immunoglobulin on the cytokine network and differentiation of the immune cells [42].To exemplify this, when immunoglobulin profiles are abnormal in RPL, we see progression of Th17 activity and reduced Treg functioning. Therefore, elevated amounts of pro-inflammatory cytokines [TNF-α, IL-17]are produced in the uterine inhospitable environment, which leads to impaired implantation and an increased chance of a miscarriage [84]. The proposed mechanisms by which dysregulated immunoglobulin responses contribute to placental dysfunction and RPL are illustrated in Fig. 2.

Proposed mechanisms by which dysregulated immunoglobulin responses contribute to recurrent pregnancy loss. Pathogenic autoantibodies such as antiphospholipid antibodies bind to placental and endothelial targets, triggering complement activation, inflammation, thrombosis, and impaired trophoblast invasion. These processes lead to placental insufficiency, defective implantation, and pregnancy loss.

## Clinical evidence linking immunoglobulins to RPL

Clinical evidence supporting the role of immunoglobulin-mediated immune dysregulation in recurrent pregnancy loss varies considerably across different biomarkers and therapeutic targets [51]. While antiphospholipid antibodies are supported by robust clinical evidence and are incorporated into international diagnostic and management guidelines, several other immunological markers, including IgG subclass profiling, Fc glycosylation, regulatory B-cell dysfunction, and non-criteria autoantibodies, remain investigational [85]. The following sections critically summarize the available evidence by distinguishing clinically established biomarkers and therapeutic approaches from emerging biomarkers and experimental mechanisms [51].

### Antiphospholipid antibodies

Antiphospholipid antibodies (aPL) represent the best-established immunological cause of recurrent pregnancy loss (RPL) and constitute the serological hallmark of antiphospholipid syndrome (APS) [86]. Obstetric APS is characterized by pregnancy morbidity associated with persistent antiphospholipid antibodies, including lupus anticoagulant (LA), anticardiolipin (aCL), and anti-β2-glycoprotein I (anti-β2GPI) antibodies [87]. These antibodies promote placental thrombosis, complement activation, endothelial dysfunction, and inflammation at the maternal–fetal interface, ultimately impairing placentation and fetal development [77].

It is important to distinguish the classification criteria for APS from clinical diagnosis [88]. The internationally accepted laboratory criteria include persistent positivity for lupus anticoagulant, anticardiolipin antibodies, and anti-β2-glycoprotein I antibodies, whereas several non-criteria antibodies (such as anti-phosphatidylserine/prothrombin and anti-annexin V antibodies) remain under investigation [87]. Although these non-criteria antibodies have been associated with adverse pregnancy outcomes in some studies, their diagnostic and therapeutic significance has not yet been fully established and they are not currently included in routine APS classification criteria [89].

Furthermore, obstetric APS encompasses a spectrum of pregnancy complications, including recurrent early pregnancy loss, fetal death after 10 weeks of gestation, and placental insufficiency leading to preeclampsia, fetal growth restriction, or preterm birth [88]. These clinical manifestations should be distinguished because their underlying pathogenic mechanisms and management strategies may differ [39]. Current international guidelines recommend low-dose aspirin combined with prophylactic heparin as the standard treatment for appropriately diagnosed obstetric APS [90, 91].

### Antinuclear antibodies

Women who have had RPL that has not been explained often test positive for antinuclear antibodies (ANA), even though they do not have an autoimmune disease [92]. While it is currently not known if there is a direct pathogenic effect from the presence of ANA, this type of antibody is considered a marker for underlying immune dysregulation. Although the precise pathogenic role of antinuclear antibodies (ANA) in recurrent pregnancy loss remains uncertain, ANA positivity has been consistently associated with an increased risk of adverse pregnancy outcomes [93]. Proposed mechanisms include immune dysregulation, altered cytokine profiles, increased natural killer (NK) cell activity, and impaired immune tolerance at the maternal–fetal interface [94]. However, current evidence supports ANA primarily as an associative biomarker rather than a direct pathogenic mediator of recurrent pregnancy loss [59]. As such, the presence of ANA is increasingly being considered as an indicator of reproductive loss due to immune causes; however, more research is needed to determine what this means clinically [95]. In addition to ANA, thyroid autoantibodies, particularly anti-thyroid peroxidase (TPO) and anti-thyroglobulin (Tg) antibodies, have also been associated with an increased risk of recurrent pregnancy loss, even in euthyroid women [96]. Current evidence suggests that these antibodies are markers of underlying autoimmune dysregulation rather than direct mediators of pregnancy loss [72]. Although levothyroxine therapy has been evaluated in euthyroid women with thyroid autoantibodies, randomized clinical trials have not consistently demonstrated improvements in live birth rates [97]. Therefore, routine levothyroxine therapy is not recommended solely on the basis of thyroid autoantibody positivity unless thyroid dysfunction is also present [91].

### Anti-prothrombin and anti-protein S antibodies

By interfering with the body’s natural anticoagulant mechanisms, which are, however, necessary to maintain the vascular structure of the placenta, anti-prothrombin and anti-protein S antibodies create a prothrombotic condition [98]. Antibodies also encourage thrombosis in small vessels due to increased tissue factor release in the placenta and decidua, which can adversely affect embryonic development at the embryonic stage, leading to possible pregnancy loss [79]. Thereby becoming significant contributors to RPL in females who test negative for classical antiphospholipid antibodies [99]. Identifying these antibodies through screening may lead to the use of targeted anticoagulant or immune-modulating treatment regimens for patients who could benefit from these options [100].

### Thyroid-related and other emerging autoantibodies

Women who have thyroid autoantibodies, such as anti-thyroid peroxidase (TPO) and anti-thyroglobulin (Tg) antibodies, may be at a greater risk of experiencing miscarriages, even if their thyroid hormone levels are normal [101]. These autoimmune markers are thought to be indicators of a broad-based autoimmune vulnerability rather than directly causing pregnancy loss [102]. In addition, newer antibody markers, such as anti-annexin V and anti-HLA, have emerged as potential contributors to RPL through immune activation and decreased placental tolerance. The identification of these newer antibodies illustrates the expanding evidence for the role of immune system dysregulation in reproductive failure [103]. Overall, while certain immunoglobulin-related biomarkers and therapeutic interventions are supported by robust clinical evidence and international guideline recommendations, several others remain investigational and require further validation before routine clinical implementation. Their current level of evidence and clinical applicability are summarized in Table 3.Table 3. Evidence hierarchy of immunoglobulin-related biomarkers and therapeutic approaches in recurrent pregnancy loss.

**Table:** 

Biomarker / Therapy	Clinical Evidence	Current Clinical Status	Evidence Level
Lupus anticoagulant	International guidelines, RCTs, cohort studies	Established	High [88]
Anticardiolipin antibodies	APS classification criteria	Established	High [89]
Anti-β2-glycoprotein I antibodies	APS classification criteria	Established	High [90]
Low-dose aspirin + heparin	Guideline-recommended for obstetric APS	Established therapy	High [92]
Antinuclear antibodies (ANA)	Observational studies	Associated marker	Moderate [96]
Anti-thyroid antibodies	Cohort studies	Emerging marker	Moderate [97]
IgG subclass profiling	Observational studies	Investigational	Moderate [62]
Fc glycosylation	Experimental and translational studies	Investigational biomarker	Low [75]
Regulatory B cells	Small clinical studies	Investigational	Low [76]
Anti-annexin V / Anti-HLA antibodies	Limited and inconsistent evidence	Experimental	Low [71]
IVIG	Mixed randomized trials and meta-analyses	Selected patients only	Moderate [49]

## Regulatory B cells and immunoglobulin homeostasis in RPL

### IL-10–producing B cells

Regulatory B lymphocytes (Bregs), which produce the cytokine interleukin-10 (IL-10), are essential to achieve immunoglobulin-mediated immune tolerance during pregnancy [104]. These Bregs help create a tolerogenic environment for the developing fetus by inhibiting pro-inflammatory immune responses and assisting in the establishment of maternal-fetal immune tolerance [105]. A reduction of both the numbers and functionality of Bregs has been reported in women with RPL, resulting in decreased levels of IL-10 and increased levels of inflammatory activity. Bregs also interact with other immune cells, including natural killer (NK) cells and regulatory T cells (Tregs), to stimulate the expansion of Tregs and inhibit the excessive cytotoxicity of NK cells [76].

### Crosstalk with Tregs and NK cells

Bregs, Tregs, and NK cells play a critical role in preserving immune equilibrium during pregnancy [83]. Malfunctioning of this network in RPL leads to a predominance of the pro-inflammatory pathways of Th1 and Th17, with elevated NK cell activity and increased generation of detrimental IgG autoantibodies [106]. The loss of maternal-fetal tolerance due to this immunological imbalance can ultimately contribute to the failure of implantation and recurrent pregnancy loss [107]. Therefore, the findings discussed provide evidence for implementing IgG-associated regulatory networks in the development of therapeutic approaches [56].

## Therapeutic implications of targeting immunoglobulin-mediated pathway

### Immunomodulatory therapies in RPL

Immunomodulatory therapy is used to restore maternal-fetal immune tolerance by correcting immune dysregulation in women experiencing recurrent pregnancy loss [108]. IVIG acts to neutralise pathogenic autoantibodies, modulate Fc receptor signalling, inhibit complement activation [78]. Treatments commonly used include intravenous immunoglobulin (IVIG), corticosteroids, low-dose aspirin, heparin, and biologics; the type of therapy selected varies based on patients’ underlying immunologic abnormalities and clinical presentations [109].

### Intravenous immunoglobulin (IVIG): Mechanisms of action

Intravenous immunoglobulin (IVIG) has been investigated as an immunomodulatory therapy for selected women with recurrent pregnancy loss, particularly those with suspected immune-mediated disease [110]. IVIG exerts multiple immunoregulatory effects, including neutralization of pathogenic autoantibodies, modulation of Fc receptor signaling, inhibition of complement activation, regulation of cytokine networks, and enhancement of regulatory immune cell function [111]. Despite these biological mechanisms, its clinical efficacy in recurrent pregnancy loss remains controversial [56]. IVIG acts to neutralize pathogenic autoantibodies, modulate Fc receptor signaling, inhibit complement activation, and regulate cytokine networks [42].In addition, IVIG reduces the cytotoxicity of NK and Th1 cells, increases the expansion of regulatory T cells, and promotes maternal-fetal immune tolerance [112]. Combined, these procedures enhance implantation, placental growth and maternal-fetal immune tolerance [108]. Several randomized controlled trials and meta-analyses have reported improved live birth rates in carefully selected patient populations; however, other studies have failed to demonstrate a significant clinical benefit [23]. Some observational studies suggest that women with elevated natural killer cell activity or autoimmune abnormalities may derive greater benefit from IVIG therapy; however, these findings remain hypothesis-generating and require validation in adequately powered prospective clinical trials [113]. However, variability among studies regarding the way participants are selected or how studies are designed limits conclusions about general therapeutic safety and efficacy [43]. The use of immunotherapy alone or through multiple combinations can act to target different pathogenic pathways that are involved in RPL [109]. The use of IVIG may improve uteroplacental perfusion, decrease inflammatory response & increase immunological tolerance when combined with low-dose aspirin or corticosteroids [78]. Anti-TNF agents have been shown to have activity in several conditions characterized by inflammation. There may be potential benefits for combination therapies, but evaluation of the risks versus benefits, as well as the need for individualized treatment, is critical [100]. Current international guidelines do not recommend the routine use of IVIG for all women with recurrent pregnancy loss because of inconsistent evidence regarding its efficacy, considerable heterogeneity in patient selection, treatment protocols, dosing regimens, timing of administration, and study endpoints [114]. Consequently, IVIG should currently be considered only in carefully selected patients within specialized clinical settings or research protocols until further high-quality randomized controlled trials establish its clinical benefit [49]. Future studies should focus on identifying biomarkers that may predict treatment response and facilitate personalized immunomodulatory therapy in carefully selected patient populations [51]. Common immunomodulatory therapies targeting immunoglobulin-mediated immune dysregulation in RPL and their mechanisms of action are summarised in Table 3.

**Table 3:** Immunomodulatory therapies targeting immunoglobulin-mediated mechanisms in recurrent pregnancy loss: mechanisms of action and current level of clinical evidence

Therapy	Mechanism	Immunological Target	Indication	Clinical Evidence and Current Recommendation
Intravenous immunoglobulin (IVIG)	Neutralises autoantibodies, modulates Fc receptors, suppresses complement activation, and enhances Treg response.	Autoantibodies, Fc signaling, cytokine networks	Immune-mediated RPL, elevated NK activity	Mixed evidence from randomized controlled trials and meta-analyses. Routine use is not recommended by current international guidelines; however, selected women with suspected immune-mediated RPL may benefit [43].
Low-dose Aspirin	Inhibits platelet aggregation, improves uteroplacental blood flow	Platelets, endothelial function	APS, thrombophilia RPL	Recommended in combination with heparin for women with obstetric APS. Evidence does not support routine use in unexplained RPL. [114].
Heparin	Anticoagulant; inhibits thrombin and complement activation	Coagulation cascade, complement system	APS-associated RPL	Strong evidence supports use in combination with low-dose aspirin for obstetric APS. No established benefit has been demonstrated in unexplained RPL without APS[78].
Corticosteroids	Suppress inflammation, reduce autoantibody production	Cytokines, immune cells	Autoimmune-associated RPL	Limited evidence. Routine use is not recommended because potential maternal and fetal adverse effects may outweigh benefits, except in selected autoimmune conditions [93].
Biologics (e.g., anti-TNF)	Target specific cytokines and inflammatory pathways	TNF-α, immune signalling	Experimental/selected inflammatory RPL	Experimental therapy. Current evidence is insufficient to recommend routine clinical use outside research settings [99].

## Patient selection and personalized treatment approaches

### Identifying immune-mediated RPL

Immune-mediated RPL is characterized by impaired maternal–fetal immune tolerance and abnormal inflammatory responses, in addition to other immunologically mediated diseases such as RPL [59]. Immune abnormalities may contribute to inflammatory injury at the maternal–fetal interface are all examples of immune mediators that contribute to RPL; all of this is caused by abnormalities in IgG production due to increased antiphospholipid antibodies and increased autoantibodies, as found in the immunobidirectional evidence and detection [80]. To establish an accurate diagnosis for RPL, the clinician must use both IgG subclasses and IgG antibodies to detect and rule out the presence of immunologically related disorders prior to giving a definitive diagnosis and establishing a tailored treatment plan for the patient [40].

### Role of immunoglobulin profiles in therapy selection

In the management of RPL, immunoglobulin profiling is an important factor in determining individualized patient treatment plans [40]. Patients who exhibit complex antibody patterns, including the presence of antiphospholipid antibodies or imbalances in IgG subclasses, may potentially respond positively to immunomodulatory therapy [109]. A patient’s specific immunological profile will dictate which treatment options, such as low-dose aspirin, heparin, corticosteroids and intravenous immunoglobulin (IVIG), would be best suited for that patient [39]. Using immunological profiling in this manner will enhance clinical decision-making and lead to an improvement in treatment outcomes [23].

### Predictors of treatment response

Several different variables contribute to how well immunomodulatory medications work to treat the immunological dysfunction that manifests through RPL. These variables include baseline immunoglobulin levels, titers of any autoantibodies present, markers of complement activation, prior obstetric history, etc. [23]. Those patients with definitive evidence of immune dysfunction are more likely to respond to targeted immunomodulatory therapies than those without obvious immune deficiencies [113]. Evaluating the dynamic changes in immunoglobulin levels during treatment may allow for better predictions of treatment response/optimizing treatment approaches [115].

## Implications in assisted reproductive technologies (ART)

The regulation of the immune system via immunoglobulins is important in assisted reproductive technologies (ART) for their influence on implantation and pregnancy [116]. Autoantibodies have been associated with recurrent implantation failure and poor in vitro fertilization (IVF) outcomes due to abnormal immunoglobulin profiles [117]. Autoantibodies can alter endometrial receptivity, interfere with trophoblast invasion, create a pro-inflammatory environment, and reduce the ability of embryos to implant [118].

In addition to the fact that changing the subclass distribution of IG4 and Fc receptor associations can also disrupt maternal-fetal immune tolerance during early implantation stages, this instability can lead to complement activation and endothelial damage, which will exacerbate the state of the uterus necessary for successful embryo development [62]. Recent advancements in the area of immune profiling, specifically the immunoglobulin assay, have shown promise as a predictive tool for identifying individuals who are at risk of experiencing ART failure.

Immunomodulatory treatments such as intravenous immunoglobulin (IVIG) might help individuals with unexplained recurrent pregnancy losses caused by an autoimmune system disorder (and the absence of other identifiable infertility factors) [119]. IVIG may be thought to produce its therapeutic effect for treating HRIF through the modulation of regulatory immune reactions via neutralisation of pathogenic autoantibodies, inhibition of excessive inflammatory responses, and restoration/recovery of immune tolerance [120]. However, clinical outcomes with IVIG vary significantly, making careful consideration regarding patient selection and consistency of treatment protocols important. Ultimately, by incorporating immunological evaluations into the overall ART treatment plan, there may be improved chances of treating reproductive health issues effectively, as well as enhancing the individualised nature of reproductive health services [121]. Although immunoglobulin-mediated immune dysregulation has emerged as a promising target for improving ART outcomes, current evidence remains heterogeneous, with considerable variability in patient selection, immunological assessment, treatment protocols, and clinical endpoints [122]. Consequently, routine use of immunomodulatory therapies, including intravenous immunoglobulin (IVIG), cannot presently be recommended for all women undergoing ART [123]. Future large-scale, well-designed randomized controlled trials and biomarker-guided precision medicine approaches are required to identify patients who are most likely to benefit from targeted immunomodulatory interventions [47].

## Challenges, controversies, and limitations

## Heterogeneity in study design

 Significant variety in study design characterizes research examining immunoglobulins in RPL including variations in patient selection criteria, definitions of RPL, timing of immunological testing, and types or doses of immunoglobulin therapy [43]. Differences in research outcomes and follow-up time make cross-trial comparisons more difficult and restrict the capacity to reach solid, broadly applicable results [124]. Immunoglobulin-based treatments for RPL have been the subject of inconsistent results in clinical trials, ranging from notable increases in live birth rates to no discernible benefit [23]. These discrepancies could be caused by disparities in treatment methods, short sample sizes, and patient heterogeneity in underlying immunological disease [113].

## Conflicting clinical outcomes

There is, therefore, a current controversy over the therapeutic efficacy of immunoglobulin therapy in practice [124]. Lack of systematic immunological analysis presents a major obstacle to immunoglobulin-center RPL studies [125]. Studies utilize different assays, cutoff values, and immunoglobulin subclasses, which are often not timely in relation to pregnancy [40]. Such heterogeneity ultimately prevents the exploration of immunological profiles that can be predictive of therapy response because it hinders reproducibility and meaningful classification of patients [43].

## Future perspectives and research directions

The immunoglobulin test should be standardized to make a more definitive diagnosis of immune-mediated RPL [40]. The difficulties in cross-study comparability include variations in test platforms, reference ranges, sampling schedules, and selection of patients [43]. Consensus guidelines on immunoglobulin subclasses, functional antibody assays and quality control procedures ought to be developed to enhance reproducibility and the ability to translate evidence into clinical practice [125] .

New immunotherapeutic strategies against RPL are aimed at sustaining fetal tolerance and altering the abnormal immune responses of the mother [126]. Examples of new measures include targeted intravenous immunoglobulin (IVIG) regimens, Fc-receptor blockage, cytokine-targeted biologic therapy and regulatory B-cell therapy [127]. Though effective randomized controlled trials are needed to determine efficacy, safety and optimal patient selection, these medicines present the possibility of a tailor-made intervention [43]. The presence of an immune profile in the routine practice of therapeutic practice can make risk categorization and RPL management customized. Immune cell interactions, autoantibody status and immunoglobulin patterns can be used to make therapeutic decisions and monitoring. But to effectively convert immunological information into effective treatment options, standardized biomarkers, cheap testing systems and physician training are required [125].

## Conclusion

Immunoglobulin-mediated immune dysregulation plays a significant role in the pathogenesis of recurrent pregnancy loss (RPL) through its effects on maternal–fetal immune tolerance, placental function, complement activation, and inflammatory signalling [128]. This review highlights the diverse roles of immunoglobulins, autoantibodies, regulatory B cells, and emerging immunological biomarkers in improving our understanding of immune-mediated pregnancy loss [47].

Among the currently available immunological markers, antiphospholipid antibodies remain the most clinically validated biomarkers and are incorporated into established diagnostic and therapeutic guidelines [88]. In contrast, other promising biomarkers, including IgG subclass alterations, Fc glycosylation patterns, regulatory B-cell dysfunction, anti-annexin V antibodies, anti-HLA antibodies, and immunoglobulin profiling, remain investigational and require further validation before routine clinical implementation [129].

Similarly, although immunomodulatory therapies such as intravenous immunoglobulin (IVIG) and selected biologic agents have demonstrated potential benefits in carefully selected patient populations, current clinical evidence remains heterogeneous and insufficient to support their routine use in all women with recurrent pregnancy loss [49]. Treatment decisions should therefore be individualized and guided by established clinical indications and current evidence-based recommendations [5, 91].

Future multicentre prospective studies, well-designed randomized controlled trials, and biomarker-guided precision medicine approaches will be essential to validate emerging immunological biomarkers, improve patient stratification, and develop personalized therapeutic strategies for women with recurrent pregnancy loss [51].

## Data Availability

No datasets were generated or analysed during the current study.
